# Genome-wide association studies of rheumatoid arthritis data via multiple hypothesis testing methods for correlated tests

**DOI:** 10.1186/1753-6561-3-s7-s38

**Published:** 2009-12-15

**Authors:** Guolian Kang, Douglas K Childers, Nianjun Liu, Kui Zhang, Guimin Gao

**Affiliations:** 1 Section on Statistical Genetics, Department of Biostatistics, The University of Alabama at Birmingham, 1665 University Boulevard, Birmingham, Alabama 35294, USA

## Abstract

Genome-wide association studies often involve testing hundreds of thousands of single-nucleotide polymorphisms (SNPs). These tests may be highly correlated because of linkage disequilibrium among SNPs. Multiple testing correction ignoring the correlation among markers, as is done in the Bonferroni procedure, can cause loss of power. Several multiple testing adjustment methods accounting for correlations among tests have been developed and have shown improved power compared to the Bonferroni procedure. These methods include a Monte Carlo (MC) method and a method of computing *p*-values adjusted for correlated tests. The objective of this study is to apply these two multiple testing methods to genome-wide association study of the Genetic Analysis Workshop 16 rheumatoid arthritis data from the North American Rheumatoid Arthritis Consortium, to compare the performance of these two methods to the Bonferroni procedure in identifying susceptibility loci underlying rheumatoid arthritis, and to discuss the strengths and weaknesses of these methods. The results show that both the MC method and *p*-values adjusted for correlated tests method identified more significant SNPs, thus potentially have higher power than the corresponding Bonferroni methods using the same test statistics as in the MC method and *p*-values adjusted for correlated tests, respectively. Simulation studies demonstrate that the MC method may have slightly higher power than the *p*-values adjusted for correlated tests method.

## Background

Genome-wide association studies (GWAS) for complex diseases involve multiple hypothesis testing. The Bonferroni procedure is commonly used to control family-wise error rate (FWER) for multiple hypothesis testing. However, the Bonferroni procedure becomes more conservative as the number of hypotheses tested increases and the test statistics are correlated [1,2]. To handle the correlation among test statistics, a permutation method [3] was proposed based on estimation of the joint distribution of test statistics. However, this approach is computationally intensive and not appropriate for GWAS. Therefore, several efficient methods that can account for the correlation among test statistics have been developed for multiple testing [1,2]. Lin [2] proposed a Monte Carlo (MC) sampling approach based on approximating the joint distribution of test statistics. This method does not require repeated analyses of simulated datasets as in the permutation method, and therefore is much less computationally demanding. Conneely and Boehnke [1] proposed a method of computing *p*-values adjusted for correlated tests (*p*_ACT) by numerical integration of the asymptotic multivariate normal distribution of the test statistics. This approach is very computationally efficient and attains even greater speed. In this study we applied three multiple testing procedures, the Bonferroni procedure, MC method, and *p*_ACT method, to GWAS of the Genetic Analysis Workshop 16 (GAW16) rheumatoid arthritis (RA) data from the North American Rheumatoid Arthritis Consortium (NARAC). We compared the performance of these three procedures by simulation studies.

## Methods

We describe the three multiple hypothesis testing procedures in the context of association studies. Suppose there are *n *individuals with *m *markers in the observed case-control data. We test *m *null hypotheses *H*_1_, *H*_2_, ..., *H*_*m *_for the *m *markers. The corresponding *p*-values are *p*_1_, *p*_2_, ..., *p*_*m*_. In the Bonferroni procedure, if *p*_*i *_≤ *α*/*m*, then *H*_*i *_is rejected (*i *= 1, ..., *m*), where *α *is the pre-set significance level. While in the Bonferroni procedure all tests are assumed to be independent, the MC method and the *p*_ACT methods described below account for dependence among test statistics by considering the joint distribution of test statistics. All the three methods can control the FWER well.

### MC method

The test statistic for the *j*^th ^marker (corresponding to hypothesis *H*_*j*_) is defined as

where , , *Y*_*i *_is the phenotypic value of individual *i *and ; *X*_*ji *_is the genotypic score of individual *i *at locus *j *and ; . If the hypothesis *H*_*j *_is true, the statistic *T*_*j *_has approximately a *χ*^2 ^distribution with *d*_*j *_degrees of freedom, where *d*_*j *_is the dimension of *U*_*j*_. For GWAS of the RA data in this article, we only consider an additive genetic model with *d*_*j *_= 1, and *X*_*ji *_= 0, 1, or 2, indicating the number of minor alleles in the genotype of individual *i *at locus *j*.

The test statistics (*T*_1_, *T*_2_, ..., *T*_*m*_) may be correlated due to linkage disequilibrium (LD) among markers. The multiple testing procedure using the actual joint distribution of (*T*_1_, *T*_2_, ..., *T*_*m*_) can be computationally intensive. The MC method provides an approach to approximate the actual joint distribution by MC sampling. The MC method defines , where  and *G*_1_, *G*_2_, ..., *G*_*n *_are independent standard normal random variables that are independent of the data, and then the method uses the joint distribution of  values to approximate the joint distribution of *T*_*j *_values based on obtaining realizations from distributions of  values by repeatedly generating the normal random samples *G*_1_, *G*_2_, ..., *G*_*n*_. Let *t*_(1) _≥ *t*_(2) _≥ ⋯ ≥ *t*_(*m*) _be the ordered observed values of the test statistics (*T*_1_, *T*_2_, ..., *T*_*m*_), and let *H*_(1)_, *H*_(2)_..., *H*_(*m*) _be the hypotheses and  are  variables corresponding to (*t*_(1)_, *t*_(2)_, ..., *t*_(*m*)_), respectively. The MC method works as a step-down procedure as follows: starting with hypothesis *H*_(1)_, the method rejects *H*_(*j*)_, *j *= 1, 2, ..., *m *and removes the corresponding marker and variable  from consideration, if , provided that *H*_(1)_, ..., *H*_(*j*-1) _have been tested and rejected. This probability is calculated based on a large number (e.g., 10,000) of realizations of the  values. We have implemented this method by using the statistical package R [4].

### p_ACT method

Suppose the test statistics **T **= (*T*_1_, *T*_2_, ⋯, *T*_*m*_) for *m *markers follow multivariate normal distribution *N*(**0**, **Σ**) asymptotically when all null hypotheses are true (i.e., no markers are associated with the disease), where **0 **is an *m*-dimensional vectors of zeros and Σ is a *m *× *m *correlation matrix. Let *p*_min _≤ *p*_(2) _≤ ⋯ ≤ *p*_(*m*) _be the ordered *p*-values calculated from the observed data. The probability of observing at least one *p*-value as small as *p*_min _is

where Z_1_, Z_2_, ⋯, Z_*m *_are random variables from the multivariate normal distribution *N*(0, Σ). Computation of *p*_*ACT *_requires integration of the multiple normal density function. The current version of the *p*_ACT method can handle integration of up to 1,000 dimensions (i.e., 1,000 markers) at one time [5]. Although any test statistics **T **with asymptotic multivariate normal distribution can be used for Eq. (2), Conneely and Boehnke [1] described the test statistic

where , . These test statistics (*T*_1_, *T*_1_, ..., *T*_*m*_) asymptotically follow multivariate normal distribution *N*(**0**, **R**), where **R **= {*r*_*jk*_}, *j *= 1, ..., *m*, *k *= 1, ..., *m*, and ; . The *p*_ACT method also works as a step-down procedure: 1) If *p*_*ACT *_<*α*, then reject the null hypothesis associated with *p*_min_, and remove *p*_min _and the marker associated with *p*_min_. 2) Let *p*_min _= *p*_(2) _in Eq. (2), change *m *into *m*-1, and repeat Step 1. Continuing in this fashion for the remaining *p*_(*j*) _until for some *p*_(*k*)_, *p*_*ACT *_= *α*, then accept all remaining hypotheses (including that associated with *p*_(*k*)_). This *p*_ACT method has been implemented in a computer program in R [6].

### Partitioning of genome-wide single-nucleotide polymorphism (SNP) data

As stated earlier, the *p*_ACT method can only handle up to 1,000 tests at a time, and the MC method can handle more than 1,000 tests but may become computationally intensive when the number of tests is very large. To apply these methods to GWAS of the RA data, we divided the whole genome into small blocks; each block includes hundreds or up to one thousand of SNPs. We assume that tests within each block are dependent, and that tests from different blocks are independent. To control the FWER at *α *for the whole genome, we apply Bonferroni procedure among blocks, that is, we assign *α*_*b *_to each block such that the sum of these *α*_*b *_equals *α *(i.e., ∑ *α*_*b *_= *α*), where *α*_*b *_is proportional to the number of SNPs within the block (i.e., block size). We applied the MC method and *p*_ACT method separately to each block and control FWER at *α*_*b *_for the tests within the block. In this study, we considered the block sizes of 100, 500, and 1,000 separately to evaluate the effect of block size on the performance of the MC method and *p*_ACT method.

### Application to RA data

The RA dataset contains 868 cases and 1,194 controls with 545,080 SNPs after removing duplicated and contaminated samples. If an individual has missing genotype at a marker, we imputed the most-frequent genotype observed in the data at that marker. We removed SNPs with minor allele frequencies (MAF) less than 0.01 and SNPs with *p*-values less than 1 × 10^-4 ^in Hardy-Weinberg equilibrium (HWE) test in controls. We did not consider SNPs on sex chromosomes. After these procedures, 515,050 SNPs remained in our analysis.

We applied the three multiple testing procedures to the GWAS of the RA dataset. First, we performed the association analysis on the RA dataset by using the test statistics in Eqs. (1) and (3) separately and obtained *p*-values for each SNP. We call these *p*-values raw *p*-values. For each test statistic, we applied the Bonferroni procedure to the *p*-values and set the nominal level of FWER as 0.05. The raw *p*-values calculated from statistic in Eq. (3) were used in the *p*_ACT method (see below). The MC method is based on the statistic in Eq. (1), and we set the number of replicates of the normal random samples *G*_1_, *G*_2_, ..., *G*_*n *_as 25,000. The *p*_ACT method is based on the test statistic in Eq. (3), and all tests are two-sided. In the *p*_ACT method we set the limit on the number of simulations or integrand values in R function "pmvnorm" as maxpts = 25,000.

### Simulation studies

To evaluate the performance of the two statistics in Eqs. (1) and (3) and of three multiple testing methods, we simulated 10,000 replicated data sets in a manner similar to Lin [2]; each data set included *N*_1 _cases and *N*_2 _controls with 100 SNPs in a chromosomal region (or one block). We considered two sets of values of *N*_1 _and *N*_2_: 1) *N*_1 _= *N*_2 _= 100, and 2) *N*_1 _= 100, and *N*_2 _= 150. For each individual, the simulated chromosomal region consisted five independent consecutive subregions. Each subregion has 20 biallelic SNPs in LD with coefficient of *r*^2 ^= 0.9 between two successive SNPs. At each SNP, HWE was assumed and MAF was 0.3. We chose one SNP in the first subregion as a disease SNP, and determined case/control status based on an additive disease model with disease prevalence of 0.1 and genotype relative risk of 2.

## Results

Table 1 shows the estimated FWER and power for the simulated datasets with nominal level of FWER = 0.05 (i.e., *α *= 0.05). The estimated FWER was calculated based on whether any SNP in any of the last four subregions was significant. The power was estimated based on whether any SNP in the first subregion is significant. In the situation *N*_1 _= *N*_2 _= 100, the two test statistics in Eqs. (1) and (3) with Bonferroni correction generated almost the same results (on FWER and power), and the MC method and *p*_ACT method also had nearly same results. In the situation *N*_1 _= 100 and *N*_2 _= 150, the test statistic in Eq. (1) had slightly higher power than that in Eq. (3), and consequently, the MC method had slightly higher power than the *p*_ACT method. In both situations, the MC method and *p*_ACT method had higher power than the Bonferroni methods using statistics in Eqs. (1) and (3), respectively.

**Table 1:** Estimated FWER and power from the simulated 10,000 replicated data sets

	*T*_*j *_in Eq. (1)		*T*_*j *_in Eq. (3)	
		
Sample size	Bonferroni	MC	Bonferroni	p_ACT
*N*_1 _= *N*_2 _= 100^a^				
FWER	0.013	0.036	0.013	0.038
Power	0.418	0.556	0.418	0.554
*N*_1 _= 100, *N*_2 _= 150				
FWER	0.023	0.054	0.022	0.053
Power	0.531	0.648	0.521	0.635

^a^*N*_1_, the number of cases; *N*_2_, the number of controls.

Table 2 reports the number of significant SNPs associated with RA on 22 chromosomes detected by the three procedures, where the overall significant level *α *across the whole genome was 0.05. By using the Bonferroni procedure, we identified 634 and 589 significant SNPs for the 22 chromosomes based on the statistics defined in Eqs. (1) and (3), respectively. The test statistic in Eq. (1) identified more significant SNPs. Table 2 also describes the number of significant SNPs on chromosomes 1, 6, 9, 16, and 22, which had more identified significant SNPs than other chromosomes. Based on the statistic in Eq. (1), the MC method identified 667, 679, and 682 significant SNPs for the 22 chromosomes when the block sizes are 100, 500, and 1,000, respectively. These numbers of identified significant SNPs are greater than the 634 identified by the Bonferroni procedure using the same statistic. Similarly, based on the statistic in Eq. (3), the *p*_ACT method identified 611, 621, and 635 significant SNPs, when the block sizes were 100, 500, and 1,000, respectively. These numbers of identified significant SNPs are also greater than the 589 identified by the Bonferroni procedure using the statistic in Eq. (3). As the block size increased, the numbers of significant SNP identified by both the MC method and the *p*_ACT method increased. The MC method using the statistic in Eq. (1) identified more significant SNPs than the *p*_ACT method using the statistic in Eq. (3).

**Table 2:** The numbers of identified significant SNPs from the RA data set

		*T*_*j *_in Eq. (1) MC				*T*_*j *_in Eq. (3) p_ACT		
				
		Block size				Block size		
		
Chromosome	Bonferroni	100	500	1,000	Bonferonni	100	500	1,000
1	24	25	25	25	22	24	23	24
6	380	392	393	413	365	372	374	377
9	21	25	21	21	19	22	20	22
16	14	15	15	14	14	14	14	14
22	17	18	19	18	14	14	17	18

1-22	634	667	679	682	589	611	621	635

We compared computing times of the MC method and *p*_ACT method. As an example, we only showed the times for chromosome 9. With block sizes of 100, 500, and 1,000, the MC method used about 6.24 hr, 2.89 hr, and 2.48 hr, while the *p*_ACT method used 0.27 hr, 0.47 hr, and 1.08 hr, respectively. The *p*_ACT method is faster than the MC method.

## Discussion

We have applied two multiple testing methods (MC and *p*_ACT), which account for correlation among tests by splitting each chromosome into smaller blocks, to the GWAS of the RA dataset and then compared the results of these methods to those of the Bonferroni procedure. Both the MC method and *p*_ACT method identified more significant SNPs than the Bonferroni procedure. The numbers of significant SNPs identified by the MC and *p*_ACT methods increased as the block size increased.

The test statistic in Eq. (3) is transformed from a traditional score statistic from a generalized linear model. The essential difference between the statistics in Eqs. (1) and (3) is that variance *V*_*j *_is estimated by different ways. Our simulation studies show that when the numbers of cases and controls are equal, the two test statistics have almost the same power, and that when the numbers of cases and controls are different, the test statistic in Eq. (1) can have slightly higher power than that in Eq. (3), and consequently, the power of the MC method can be slightly higher than that of *p*_ACT method. Our simulation studies were only based on additive model, small sample sizes, and small number of SNPs. More extensive simulation studies are necessary in the future research.

In our analysis, we divided the whole genome into blocks with a fixed number of SNPs. We only accounted for LD within each block, and we assumed independence between tests from different blocks by ignoring LD between blocks. This assumption can cause loss of power. To avoid losing power, each entire chromosome may be treated as a block. However, the MC method will become computationally intensive or infeasible, and the *p_*ACT cannot handle more than 1,000 SNPs in each block. Another possible solution is to split each chromosome into blocks according to LD pattern, to group a set of consecutive SNPs in strong LD into one block and to ignore weak LD between blocks. As described earlier, the larger the block size we select, the higher the power we can obtain. This is an issue we will pursue in the future. Also we did not consider population stratification, which may cause spurious false-positive results.

## List of abbreviations used

FWER: Family-wise error rate; GAW16: Genetic Analysis Workshop 16; GWAS: genome-wide association studies; HWE: Hardy-Weinberg equilibrium; LD: Linkage disequilibrium; MAF: Minor allele frequency; MC: Monte Carlo; NARAC: North American Rheumatoid Arthritis Consortium; *p*_ACT: *p*-Values adjusted for correlated tests; RA: Rheumatoid arthritis; SNP: Single-nucleotide polymorphism.

## Competing interests

The authors declare that they have no competing interests.

## Authors' contributions

GK did all simulation studies and data analysis, and drafted and revised the manuscript. DKC participated in the design of the study. NL and KZ participated in the design of the study and revised the manuscript. GG directed the study and partially drafted and revised the manuscript. All authors read and approved the final manuscript.
